# Photosonochemical catalytic ring opening of α-epoxyketones

**DOI:** 10.1186/1860-5397-3-2

**Published:** 2007-01-27

**Authors:** Hamid R Memarian, Ali Saffar-Teluri

**Affiliations:** 1 Department of Chemistry, Faculty of Science, University of Isfahan, 81746-73441, Isfahan, Iran

## Abstract

The combination of ultrasound and photochemical methods has been used for the catalytic ring opening of α-epoxyketones by 1-benzyl-2,4,6-triphenylpyridinium tetrafluoroborate (NBTPT) as photocatalyst in methanol. Sonication of these compounds in the presence of NBTPT did not result in the opening of epoxide ring, but the use of ultrasound increased the rate of photoreaction.

## Background

The advantages of ultrasound-assisted chemical reactions include higher yields, shorter reaction times and milder reaction conditions when compared with classical methods. [1–5] The effect of ultrasound has mostly been shown by increasing the yields of reactions and in some cases changing the ratio of products formed. The most important effects of ultrasound arise from acoustic cavitation: formation, growth, and implosive collapse of bubbles in the liquid by passing ultrasonic waves through this medium. [3,6] The implosive collapse of the bubble generates localized hot spots through adiabatic compression or shock wave formation within the gas phase of the collapsing bubble. This leads to development of temperatures up to 5000 K and high pressures of 1800 atm and cooling rates in excess of 10^10^ K/s within the cavities during their collapse. [3,6] In all of these reactions it was found that ultrasound accelerates the reactions. [7–17]

It is well known that the substituted pyridinium cations are good electron acceptors. [18] Garcia and coworkers have used *N*-alkyl-2,4,6-triphenylpyridinium tetrafluoroborate as photosensitizer in the photochemical cyclization of 5-methyl-4-hexenoic acid to the corresponding γ-lactone. [19] In our recent study, we have used 1-benzyl-2,4,6-triphenylpyridinium tetrafluoroborate (NBTPT) as photocatalyst in a highly diastereoselective ring opening of α-epoxyketones in acetone solution with the formation of 1.3-dioxolanes. [20]

Ring opening of epoxides and α-epoxyketones in the presence of various nucleophiles has received considerable attention in recent years, partially owing to current interest in single electron transfer (SET) process and also because of potential application in organic synthesis. Such reactions have been recognized as important processes not only in thermal but also in photochemical transformations. Single electron transfer (SET) induced ring opening reactions of epoxides and α-epoxyketones have demonstrated C-C and C-O bond cleavages through photo-induced electron transfer by various electron donors such as triethylamine (TEA), [21] tribenzylamine (TBA) [20] and 1,3-dimethyl-2-phenylbenzimidazoline (DMPBI) [22–24] or thermally induced single electron transfer by electron donating compounds such as samarium diiodide, [25] tributyltin hydride [26] and bis(cyclopentadienyl)titanium(III) chloride. [27] Ring opening reactions of epoxides and α-epoxyketones have also occurred thermally or photochemically by the presence of various electron acceptors. These reactions have been observed thermally by ceric ammonium nitrate (CAN), [28–29] 2,3-dichloro-5,6-dicyano-*p*-benzoquinone (DDQ) [30] and iron(III) chloride [31] or photo-induced electron transfer reactions by dicyanoanthracene (DCA), [32–33] tetracyanoethylene (TCNE) [34–35] and 2,4,6-triphenylpyrilium tetrafluoroborate. [36–40] In the case of C-C bond cleavage, the generated intermediates from epoxide radical cations have been trapped by molecular oxygen to form trioxolane derivatives [32–34] or by dipolarophiles to form various tetrahydrofurans and dihydrofurans. [41] In the absence of appropriate dipolarophiles, *cis*/*trans* isomerization of the epoxide ring has been observed. [42] The cleavage of C_α_-O or C_β_-O bonds has been confirmed either by rearrangement to carbonyl compounds [43–50] or by nucleophilic attack of appropriate reagents. [36,39,51]

Recently, we have reported on the photocatalytic ring opening of α-epoxyketones **1a-f** and 2,4,6-triphenylpyrilium tetrafluoroborate (TPT) as photocatalyst in methanol, [37] cyclohexanone, [38] acetone [39] and acetic acid solutions. [40]

Our recent study with 1-benzyl-2,4,6-triphenylpyridinium tetrafluoroborate (NBTPT) **2** as weaker electron-acceptor compared with TPT (nitrogen *vs*. oxygen) for highly diastereoselective ring opening of α-epoxyketones in acetone [20] leads us to investigate simultaneous irradiation of ultrasound and UV-light for catalytic ring opening of α-epoxyketones **1a-f** in the presence of this photocatalyst in methanol. The main goal of the present work was to elucidate the effect of both irradiation sources separately or together on the rate of photocatalytic ring opening of α-epoxyketones and also the electron-acceptor ability of NBTPT on the diastereoselectivity of reaction.

## Results and Discussion

**Scheme 1:**
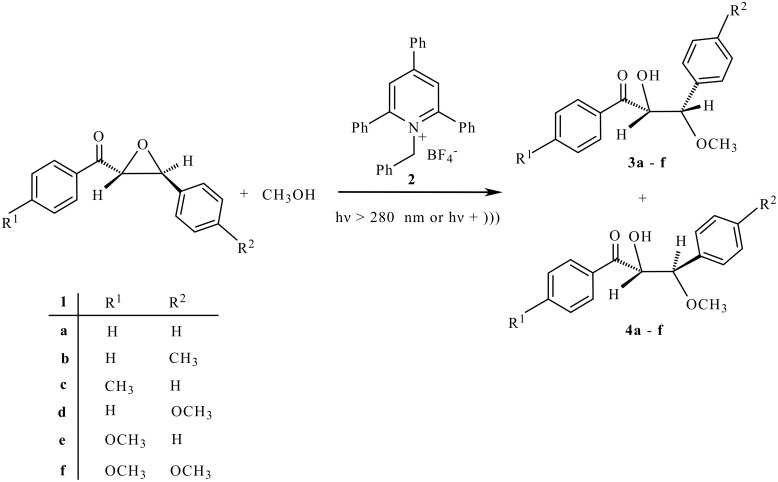
Ultrasound-assisted photocatalytic ring opening of α-epoxyketones.

Photo-induced reactions of α-epoxyketones **1a-f** with **2** in methanol solution produced a mixture of MeOH-adducts **3a-f** and **4a-f** (Scheme 1). It should be noted that sonication of the mixture of **1a-f** with **2** alone did not result in the opening of epoxide ring. The results are summarized in Table 1.

**Table 1:** Photochemical and photosonochemical reactions of **1a-f** catalyzed by **2** in methanol solution.^I^

Compound	Irradiation time (h)	Yield (%)^II^	**3**/**4**^III^	**3** %	**4** %

**1a** + *hν*	11	88	1 : 2.0	33.3	66.7
**1a** + *hν* +)))	7	90	1 : 2.1	32.3	67.7
**1b** + *hν*	2.5	99	1.2 : 1	54.5	45.5
**1b** + *hν* +)))	1.5	99	1.2 : 1	54.5	45.5
**1c** + *hν*	10	85	1 : 3.7	21.3	78.7
**1c** + *hν* +)))	9	87	1 : 3.4	22.7	77.3
**1d** + *hν*	2.15	98	1.1 : 1	52.4	47.6
**1d** + *hν* +)))	1.10	100	1.1 : 1	52.4	47.6
**1e** + *hν*	9	89	1 : 4.8	17.2	82.8
**1e** + *hν* +)))	5	90	1 : 4.8	17.2	82.8
**1f** + *hν*	2	99	1 : 1.1	47.6	52.4
**1f** + *hν* +)))	1.05	99	1 : 1.2	45.5	54.5

^I^ [**1a-f**] = 0.04 M, [**2**] = 0.004 M, corresponding to a molar ratio of 10:1. ^II^Based on consumed **1a-f**. ^III^The ratios have been determined by comparison of the integral ratios of the hydrogen on C-2.

**Scheme 2:**
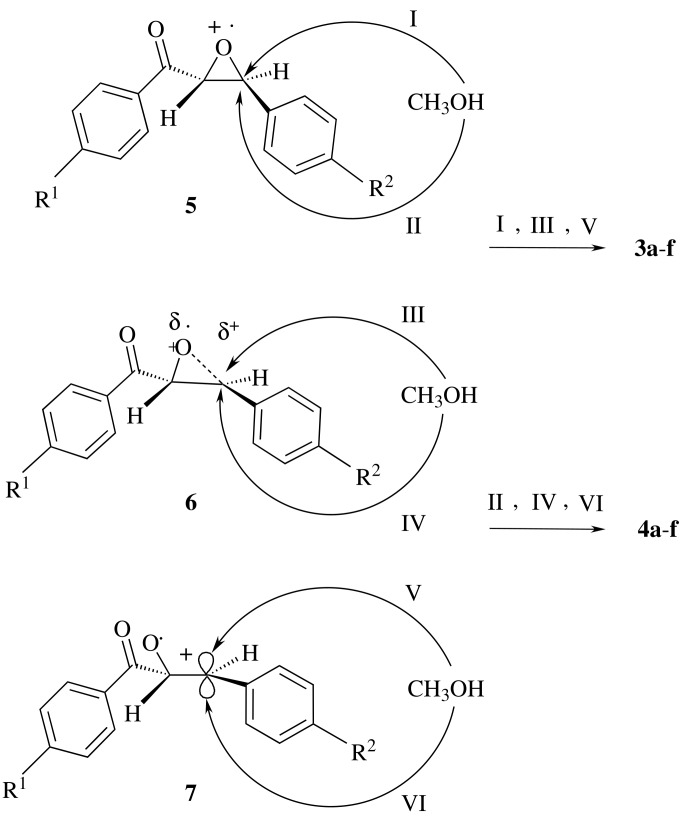
Possible intermediates involved in the ring opening of α-epoxyketones.

Comparison of the data presented in Table 1 indicates that (i) catalytic ring opening of α-epoxyketones considered in this study was accelerated by simultaneous irradiation of ultrasound and UV-light and (ii) the rate of the ring opening of α-epoxyketones in the presence of **2** is also dependent on the additional substituent on the phenyl ring. Whereas the electron donating groups (*p*-methyl and *p*-methoxy) on the phenyl ring directly attached to the epoxide ring facilitate the rate of photocatalytic ring opening of α-epoxyketones **1b**, **1d** and **1f**, the same substituents on the phenyl ring of the benzoyl group (**1c** and **1e**) have a smaller effect. In the cases of **1b** and **1d**, the ratios of the diastereomeric photoproducts are inversed. We have proposed the involvement of three different intermediates **5–7** in the photocatalytic ring opening of α-epoxyketones by TPT as strong electron acceptor due to complete electron transfer from α-epoxyketones to excited TPT (Scheme 2). A comparison of the ultra-violet data of **1a-f** with NBTPT presented in Table 2 shows that under the reaction conditions – irradiation at λ ≥ 280 nm and a molar ratio of **1a-f** : NBTPT (10 : 1) – NBTPT is excited selectively. Therefore, the same intermediates should be involved in our study.

**Table 2:** UV absorption λ_max_ and molar extinction coefficients (ε)of **1a-f** and NBTPT in CH_2_Cl_2_

Compounds	λ_max_ (nm), ε (l mol^-1^ cm^-1^)

**1a**	262 (18053), 320 (250)
**1b**	267 (17186), 324 (405)
**1c**	264 (36037), 340 (sh)
**1d**	270 (16418), 328 (580)
**1e**	277 (32570)
**1f**	286 (21897)
NBTPT	240 (8789), 313 (29448)

**Scheme 3:**
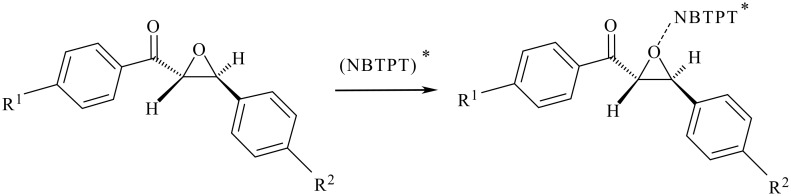
Possible formation of a complex involved in reaction in acetone.

The interesting point in the present work is that in contrast to the results obtained by reaction of **1a-f** and **2** in acetone solution, which leads to the highly diastereoselective formation of 1,3-dioxolanes, diasteroselective formations of α-hydroxy-β-methoxyether derivatives **3** and **4** have been only observed in some cases of photocatalytic ring opening of **1a-f** and **2** in methanol solution. The observed high distereoselectivity by reaction of **1a-f** and **2** in acetone solution has been explained by the involvement of a complex (**1a-f**... NBTPT*) instead of the intermediates **5–7** for the nucleophilic attack of acetone (Scheme 3). [19]

**Scheme 4:**
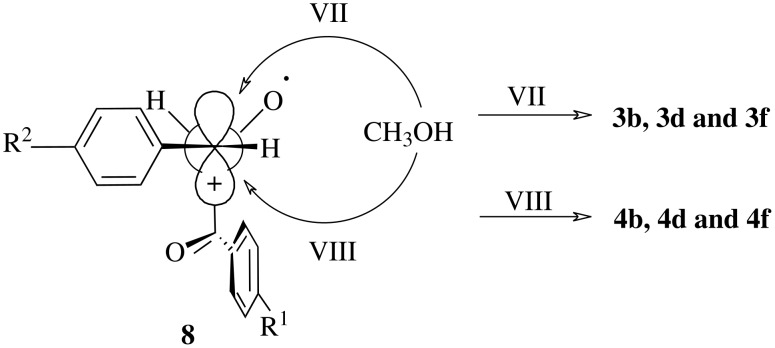
Interaction of oxygen lone pair of carbonyl group with carbocation center.

Due to the polar nature of methanol compared with acetone, we should expect a complete electron transfer from **1a-f** to photoexcited **2** under the formation of one of the intermediates **5–7**, depending on the location of the additional substituent on the parent molecule **1a**. This argument is supported by the effect of the nature of substituents on the rate of reaction and the diastereomeric ratios of products. This leads us to assume that the inductive effect of the *p*-methyl group and the resonance effect of the *p*-methoxy group on the phenyl ring directly attached to the epoxide ring increase the contribution of the intermediates **6** and **7** because of the stabilization of carbocation or carbocation-like centers. The more stable conformer of the intermediate **7** can be formed through interaction of oxygen lone pair of carbonyl group with carbocation center (Scheme 4, intermediate **8**). This leads to the preferred nucleophilic attack of methanol to the carbon atom at the less hindered site (VII) to form the *cis*-isomers **3b** and **3d** (Scheme 4). On the other hand, the intermediates e.g. **5** and **6** is proposed for **1a**, **1c** and **1e**, in which the phenyl ring attached to the epoxide ring is not bearing such electron donating groups on the phenyl ring directly attached to the epoxide ring.

The expanded part of ^1^H NMR spectra of photoproducts **3a-f** and **4a-f** (hydrogen located on C-2) shows exactly the ratios of diastereomeric products and is provided [see Supporting Information File 1].

Concerning the effect of ultrasound on the rate of ring opening of α-epoxyketones, we propose that increasing the rate of reaction is caused by efficient mass transfer of the reaction mixture by sonication. Also the electron transfer between the active species in this homogeneous system using sonication occurs faster than the system without sonication. Whereas the use of ultrasound accelerates the rate of photocatalytic ring opening of α-epoxyketones **1a-f**, the ratios of diastereomeric photoproducts have not been changed too much using ultrasound irradiation.

**Figure 1:**
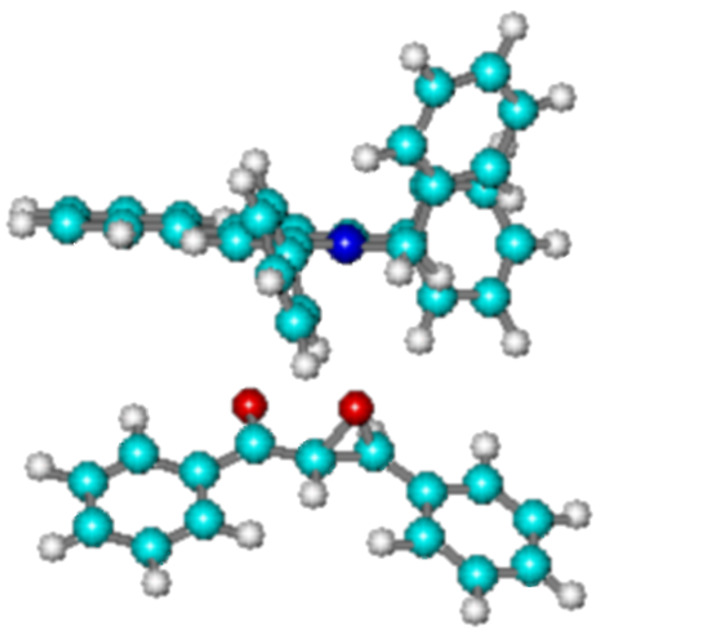
The *semi-empirical* PM3 calculations for interaction of **1a** with NBTPT.

In order to explain the results obtained, we have compared the results of the *semi-empirical* PM3 calculations on the complexes of **1a-f** + **2** in the ground state with those of α-epoxyketones **1a-f** alone (Figure 1). The data obtained are presented in Table 3.

**Table 3:** Mulliken electric charges [52–53] of the epoxide ring atoms of **1a-f** alone and the complexes of α-epoxyketones **1a-f** with **2** in the ground state obtained from quantum mechanical PM3 calculations

**1a-f**				**1a-f** + **2**		

	C-2 (C_α_)	C-3 (C_β_)	O	C-2 (C_α_)	C-3 (C_β_)	O

**1a**	-0.082	0.082	-0.229	-0.065	0.098	-0.304
**1b**	-0.083	0.084	-0.232	-0.067	0.102	-0.306
**1c**	-0.080	0.080	-0.231	-0.061	0.093	-0.304
**1d**	-0.084	0.089	-0.233	-0.063	0.101	-0.305
**1e**	-0.083	0.082	-0.232	-0.066	0.098	-0.306
**1f**	-0.083	0.088	-0.234	-0.067	0.103	-0.302

These data shows that the electric charges of atoms in the epoxides ring in the complexes of **1a-f** + **2** compared with **1a-f** alone have been increased. The increasing of the charges of oxygen and C-3 (C_β_) show that C_β_-O bond has tendency for cleavage. This tendency for the complexes of **1b**, **1d** and **1f + 2** is increased by the presence of the donor groups such as *p*-methyl and *p*-methoxy, because the charge of C-3 (C_β_) in the complexes of **1b**, **1d** and **1f + 2** is more positive than other complexes. Therefore, the nucleophilic attack of methanol to the C-3 (C_β_) in the complexes of **1b**, **1d** and **1f + 2** is faster than the others. This leads to faster ring opening of these compounds.

## Conclusion

From the results of this work and our previous work concerning photocatalytic ring opening of α-epoxyketones, it follows that ultrasound alone does not effect the epoxide ring opening. On the other hand, ultrasound can seriously affect photocatalytic ring opening of α-epoxyketones predominantly because of the efficient mass transfer of the reactants and the excited state of NBTPT. The higher yields and shorter reaction times are advantages of this method.

## Experimental

α-Epoxyketones **1a-f** and 1-benzyl-2,4,6-triphenylpyridinium tetrafluoroborate **2** were prepared according to the reported procedures. [54–56] Methanol was purchased from Merck and distilled before use. The ultrasonic device used was an UP 400 S instrument from Dr. Hielscher GmbH. A S3 immersion horn emitting 24 kHz ultrasound at intensity levels tunable up to maximum sonic power density of 460 W cm^-2^ was used. Sonication was carried out at 100 % (maximum amplitude 210 μm). A 3 mm long sonotrode (maximum immerse depth of 90 mm) was immersed directly into the reaction mixture. UV irradiations were performed using a 400 W high pressure mercury lamp from Narva with cooling of samples in Duran glass. ^1^H NMR spectra of the mixture of photoproducts were measured in CDCl_3_ solutions containing tetramethylsilane (TMS) as internal standard on a Bruker drx-500 (500 MHz). Preparative layer chromatography (PLC) was carried out on 20 × 20 cm^2^ plates coated with 1 mm layer of Merck silica gel PF_254_ prepared by applying the silica as a slurry and drying in air. All products are known and their spectral data have been reported earlier. [37]

### General procedure for the photocatalytic ring opening of 1a-f by NBTPT

A solution of a 0.8 mmol of **1a-f** in 20 cm^3^ methanol (c = 0.04 M) and 0.08 mmol of **2** (c = 0.004 M) was irradiated (λ ≥ 280 nm) for the times given in Table 1. Then, the solvent was evaporated and the mixture of photoproducts was isolated by PLC.

### General procedure for the photosonocatalytic ring opening of 1a-f by NBTPT

A solution of a 0.8 mmol of **1a-f** in 20 cm^3^ methanol (c = 0.04 M) and 0.08 mmol of **2** (c = 0.004 M) was sonicated and irradiated (λ ≥ 280 nm) simultaneously for the times given in Table 1. Then, the solvent was evaporated and the mixture of photoproducts was isolated by PLC.

## Supporting Information

File 1 Supporting materials. comparison of the integral ratios of the hydrogen on C-2 for **3a-f** and **4a-f**.
