# Supplementary material for: Photosonochemical catalytic ring opening of α-epoxyketones
Source: Beilstein J Org Chem. 2007 Jan 27;3:2. doi: 10.1186/1860-5397-3-2 (PMC1810301; doi:10.1186/1860-5397-3-2)
Supplement: File 1 — Supporting materials. comparison of the integral ratios of the hydrogen on C-2 for 3a-f and 4a-f. [file Beilstein_J_Org_Chem-03-02-s001.doc]

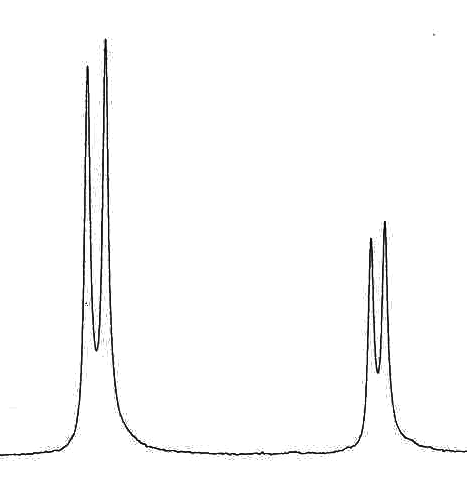

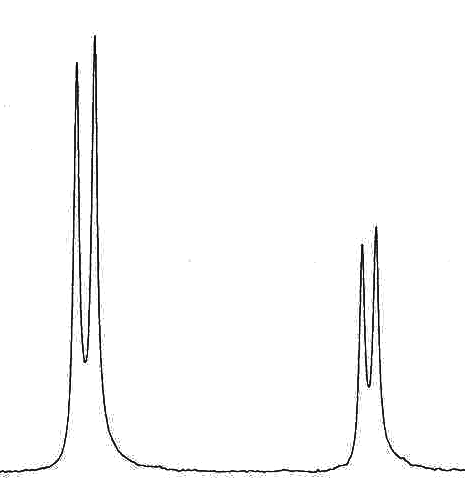

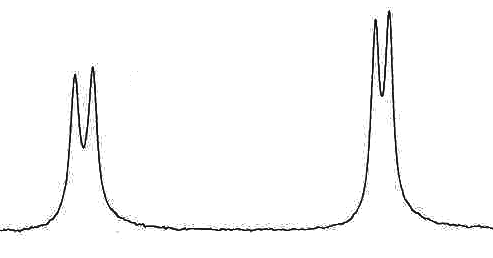

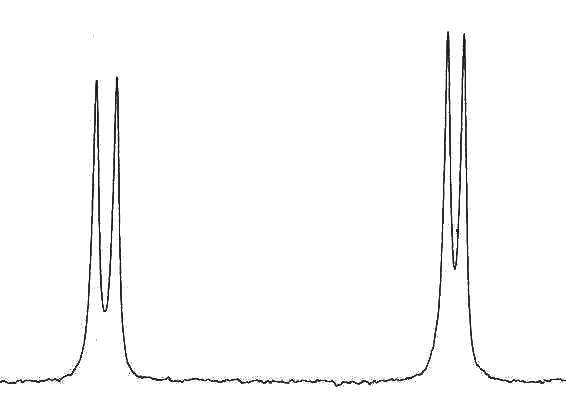


| **a** |
| --- |
| **b** |
| **c** |
| **d** |
| **e** |
| **f** |


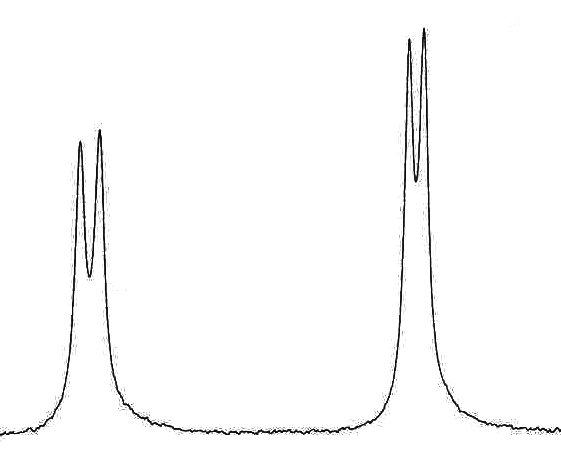


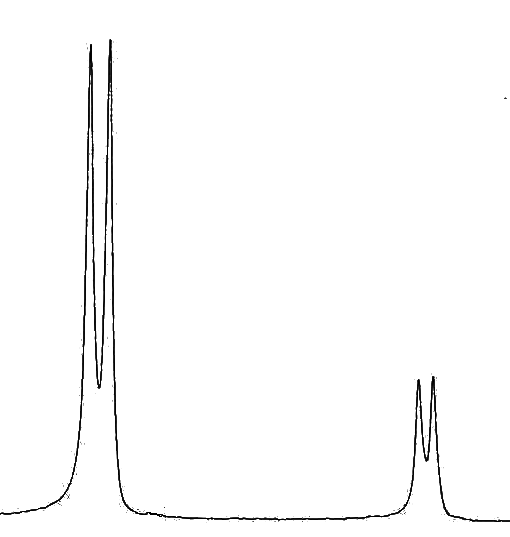

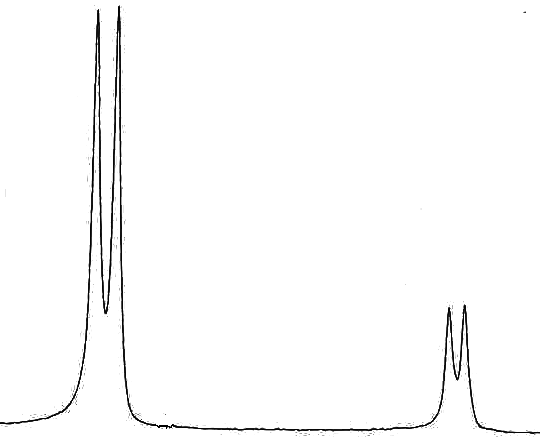


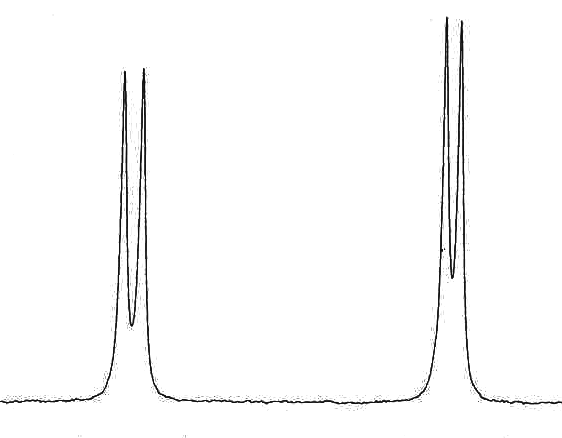


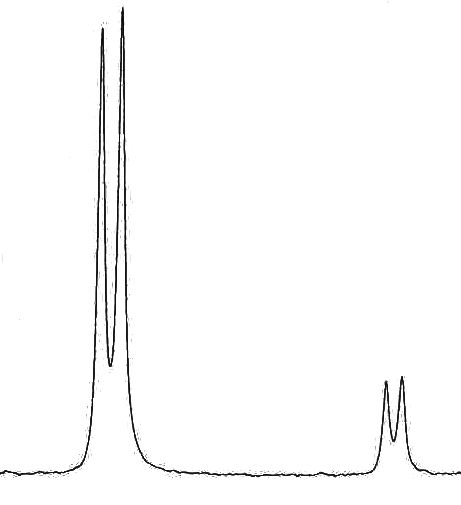

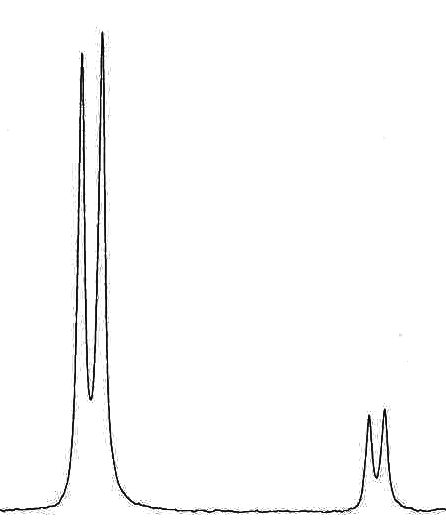


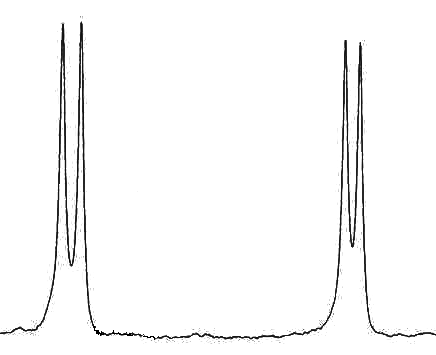


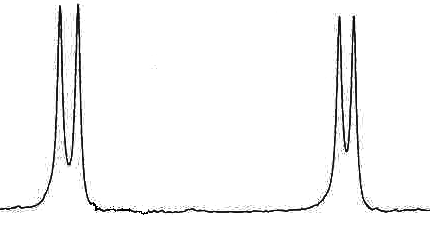


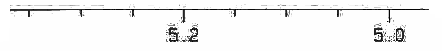

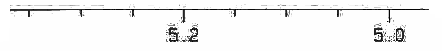


**Figure.** The comparison of the integral ratios of the hydrogen on C-2 for **3a-f** and **4a-f**; *hν* + ))) (right) and *hν* (left).
